# Astroglial Cells: Emerging Therapeutic Targets in the Management of Traumatic Brain Injury

**DOI:** 10.3390/cells13020148

**Published:** 2024-01-12

**Authors:** Wojciech Czyżewski, Marek Mazurek, Leon Sakwa, Michał Szymoniuk, Jennifer Pham, Barbara Pasierb, Jakub Litak, Ewa Czyżewska, Michał Turek, Bartłomiej Piotrowski, Kamil Torres, Radosław Rola

**Affiliations:** 1 Department of Didactics and Medical Simulation, Medical University of Lublin, 20-954 Lublin, Poland; kamiltorres@wp.pl; 2 Department of Neurosurgery and Pediatric Neurosurgery, Medical University of Lublin, 20-954 Lublin, Poland; marekmazurek@hotmail.com (M.M.); rola.radoslaw@gmail.com (R.R.); 3 Student Scientific Society, Kazimierz Pulaski University of Radom, 26-600 Radom, Poland; sakwus@gmail.com; 4 Student Scientific Association, Department of Neurosurgery and Pediatric Neurosurgery, Medical University of Lublin, 20-954 Lublin, Poland; michmatsz@gmail.com; 5 Student Scientific Society, Medical University of Lublin, 20-954 Lublin, Poland; jennifer.pham@ucla.edu (J.P.); michal.turek1997@gmail.com (M.T.); 6 Department of Dermatology, Radom Specialist Hospital, 26-600 Radom, Poland; barbarapasierb95@gmail.com; 7 Department of Clinical Immunology, Medical University of Lublin, 20-954 Lublin, Poland; jakub.litak@gmail.com; 8 Department of Otolaryngology, Mazovian Specialist Hospital, 26-617 Radom, Poland; czyzewska.ewa@hotmail.com; 9 Institute of Automatic Control and Robotics, Warsaw University of Technology, 00-661 Warsaw, Poland; bartlomiej.piotrowski.dokt@pw.edu.pl

**Keywords:** astroglia, astrocytes, traumatic brain injury

## Abstract

Traumatic Brain Injury (TBI) represents a significant health concern, necessitating advanced therapeutic interventions. This detailed review explores the critical roles of astrocytes, key cellular constituents of the central nervous system (CNS), in both the pathophysiology and possible rehabilitation of TBI. Following injury, astrocytes exhibit reactive transformations, differentiating into pro-inflammatory (A1) and neuroprotective (A2) phenotypes. This paper elucidates the interactions of astrocytes with neurons, their role in neuroinflammation, and the potential for their therapeutic exploitation. Emphasized strategies encompass the utilization of endocannabinoid and calcium signaling pathways, hormone-based treatments like 17β-estradiol, biological therapies employing anti-HBGB1 monoclonal antibodies, gene therapy targeting Connexin 43, and the innovative technique of astrocyte transplantation as a means to repair damaged neural tissues.

## 1. Introduction

Astrocytes, characterized by their heterogeneity, play a multifaceted role essential to cerebral function. These cells are instrumental in maintaining ion homeostasis, modulating the clearance of neurotransmitters, and regulating cerebral blood flow and water dynamics. Additionally, they are pivotal in the upkeep of synaptic structures and significantly contribute to the integrity of the blood-brain barrier [1,2]. Traumatic brain injury (TBI) is defined as brain damage caused by external mechanical force and it is the leading cause of death and disability especially in children and young adults [3,4,5]. It concerns approximately 64–74 million people annually [6]. In the United States alone, approximately 1.7 million people experience TBI, and over 5.3 million suffer from a trauma-related disability [7] reaching a 3% mortality rate [8]. This results in costs of roughly 56–221 billion US $ which are spent on diagnosis, treatment, and rehabilitation of patients with TBI each year [9]. The global incidence of TBI from any cause and all severity is estimated at 939 per 100,000 people. Brain injuries are traditionally classified by the Glasgow coma scale (GCS) according to their severity into mild (13–15 GCS points ), moderate (12–9 GCS points), and severe (<9 GCS points) [10,11]. The first group includes the least serious injuries and accounts for 80–90% of the total. They are usually caused by blunt, non-penetrating head trauma. Symptoms are usually transient and not very specific. They may include headache, mild cognitive symptoms, memory problems, nausea, and vomiting [4,6]. Moderate and severe TBI are associated with greater force of the trauma and their effect are often visible in imaging tests. The most severe of the groups is also associated with a very high mortality rate, both in the acute phase and later, reaching 35–75% [5,12,13]. However, despite its much lower prevalence, it is moderate and severe TBI that pose a serious problem for medicine. It is estimated that they cause approximately 90% of total TBI medical costs [14]. Many efforts have been implemented to improve the prognosis of patients as much as possible; however, no specific neuroprotective measures have been identified that can affect the mortality of patients after TBI on a broad scale [15]. Current guidelines for the management of trauma patients are primarily aimed at reducing secondary brain damage caused by a cascade of physiological processes that are the body’s response to injury. For this reason, it is so important to properly understand these relationships. As studies have shown, astrocytes play a key role in this process through the mechanism of reactive astrogliosis. According to preceding studies, restoration and regeneration following brain trauma is as complex as it is challenging due to various aspects [16]. Numerous cellular groups and biochemical compounds participate in this process, among which astrocytes are a key component. During the acute phase, injury results not only from the direct impact of external force upon the body but also from the reactive responses of astrocytes and microglia cells. These cells undergo alterations in their transcriptional and morphological profiles, leading to the initiation of both pro-inflammatory and anti-inflammatory processes. These physiological adaptations are designed to facilitate neuronal regeneration and the clearance of damaged tissue [17,18]. Upon exposure to specific stimuli, frequently originating from central nervous system (CNS) injuries or pathologies, astrocytes undergo a significant transformation into a reactive state. This process, termed “reactive astrogliosis”, is characterized by substantial changes in gene expression, cellular morphology, and functional dynamics [19]. Multiple theoretical frameworks have been proposed to elucidate the mechanisms underlying astrocyte responses to cerebral injury. Confronted with brain trauma, astroglial cells exhibit notable alterations in both functionality and phenotypic characteristics [20,21,22]. Although this reactive state of astrocytes can be conducive to healing and recovery, exemplified by functions such as stabilizing the blood-brain barrier (BBB), facilitating neurogenesis, and secreting neurotrophic compounds, it also has the potential to produce adverse effects. A significant consequence is the formation of a glial scar, traditionally regarded as an impediment to CNS repair. This scar, emerging from the collective response of reactive astrocytes, is a primary factor exacerbating neuronal regeneration. The fibrotic scar tissue not only presents a physical and chemical barrier but also disrupts the process of synaptogenesis [23,24]. Moreover, dysregulated inflammatory responses from activated astrocytes can result in neuronal harm [25]. The upregulation of cytokines and inflammatory mediators hinders synaptic regeneration and induces hypoxia, consequently leading to neuronal ischemia. Nevertheless, the precise nature of the astrocytic response to central nervous system (CNS) trauma remains elusive [26]. In this review, we aim to expound on the role of astrocytes and their reaction to CNS injuries, as well as to discuss the current therapeutic strategies targeting astrocytes in various neurological disorders, with a particular focus on trauma-related conditions.

## 2. Role of Astroglia in Brain Injury

### 2.1. Blood Brain Barrier

One of the influences of astrocytes on the homeostasis of the CNS is their effect on the blood-brain barrier. Astrogliosis acts protectively on surrounding neural tissue through the maintenance of the blood-brain barrier (BBB), repair of damaged tissues, production of neuroprotective compounds, and mediation of inflammation [22,27,28]. This process is possible because of the communication with endothelial cells [29,30,31]. Astrocytes also oversee the functioning of the BBB through specialized astrocytic extensions known as “endfeet” that are equipped with the potassium channel Kir 4.1 and Aquaporin-4, which play a crucial role in regulating ion and water balance, contributing to BBB integrity [32].

In the event of insult to the brain, astrocyte-derived factors possess a key role in recovery and disruption of the blood-brain barrier. The astrocyte-derived factors that influence vascular permeability encompass vascular endothelial growth factors, glutamate, matrix metalloproteinases, nitric oxide, and endothelin-1. These elements heighten the permeability of the blood-brain barrier, ultimately leading to its disruption. Conversely, astrocyte-derived protective elements consist of angiopoietin-1, glial-derived neurotrophic factor, insulin-like growth factor-1, sonic hedgehog, retinoic acid, and apolipoprotein E. These components mitigate blood-brain barrier permeability, promoting the restoration of its functionality [33].

### 2.2. Immunological Response

Activated astrocytes are important regulators of immune response. In response to damage, astroglial cells release neurotrophic factors that act to protect the brain [33]. For example, brain-derived neurotrophic factor (BDNF) secreted by astroglial cells has been shown to combat drug-related neurotoxic effects and neuronal degeneration associated with normal aging. Suboptimal levels of BDNF have been associated with neuronal death [34,35]. Another neuroprotective contribution is through the release of 17β-estradiol (E2) compounds from activated astrocytes [36,37]. E2 is important in mediating inflammatory responses and in reducing microglial activation in the brain [38,39]. Overactivation of microglia can lead to a feedback loop which induces more reactive astrogliosis and further inflammatory damage as a result [40,41,42]. Also, astrocytes function as an inhibitor of neuroinflammation through various physiological pathways such as TGF-β signaling or estrogen receptor signaling pathway [43,44]. Even though astroglia can downregulate inflammatory responses, its activation can also increase inflammation. Studies have found that activated astrocytes are associated with higher amounts of inflammatory markers [45,46]. Cytokines released from astrocytes also promote inflammation through microglial cells after traumatic brain injury [47,48]. Additionally, inflammation driven by the interaction between astrocytes and microglia can become neurotoxic to neural tissue [49,50,51]. In response to injury, astrocytes can release neurotoxic levels of neurotransmitters such as NO or glutamate, leading to neuronal death [27,52].

Glial cell necrosis mitigates cell’s functionality what triggers the release and subsequent accumulation of molecules known as Damage-Associated Molecular Patterns (DAMPs), such as intracellular ions, nucleic acids, high mobility group box 1 protein (HMGB1), heat shock protein 72 (Hsp72), HA and ATP. These DAMPs activate immune receptors, notably Toll-Like Receptors (TLR), Receptors for Advanced Glycation End Products (RAGE) or purinergic receptors on myeloid cells encompassing macrophages, glial cells, dendritic cells, and astrocytes which cause their activation as well as inflammasome assembly (NALP1) that advocates generation of cytokines as IL-18 or IL-1Β [53].

Upon activation, resident microglia undertake cleanup of cellular debris, restore the integrity of the damaged blood-brain barrier, and aim to deliver essential nutrients requisite for neuronal cells. However, microglia exhibit a high degree of flexibility, and their role in a non-infectious immune response is determined by their activation status, the extent of the damage, interplay with adjacent cells, and the makeup of the immune cells that have infiltrated the area. 

**Figure 1:**
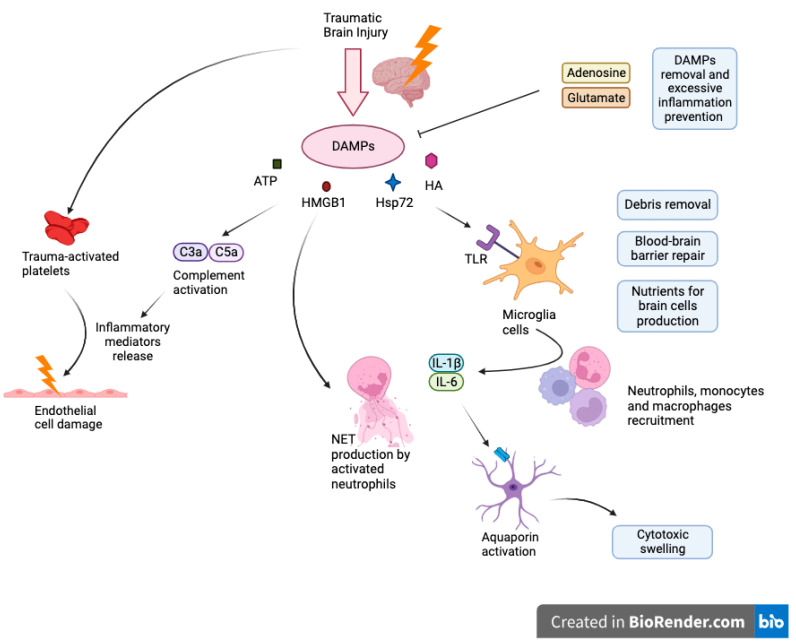
Cascade of neuroinflammation in Traumatic Brain Injury: DAMPs, glial activation, and systemic consequences.

Further release of proinflammatory cytokines such as IL-1β and IL-6 facilitates the recruitment of peripheral immune cells into the site of injury. When neutrophils traverse the BBB, they exacerbate leukocyte activation, proinflammatory cytokines levels, and incite brain tissue swelling Figure 1.

Activated neutrophils generate NETs (neutrophil extracellular traps) and potentiate inflammation. HMGB1 is implicated in the initiation of NET formation and neuroinflammation, primarily through the secretion of cytokines including IL-1β and IL-6. Astroglia, upon stimulation by cytokines such as IL-1β, chemokines, complement, and ROS engender immune signals, further recruiting neutrophils and precipitating a widespread cytokine release [54]. 

This activation cascade also results in the synthesis of proinflammatory cytokines and the facilitation of antigen presentation by antigen-presenting cells (APCs) to effector cells encompassing T and Natural Killer (NK) cells. 

Trauma-induced activation of platelets and the coagulation cascade facilitates the release of pro-inflammatory mediators and microvesicles containing DAMPs from platelets and neutrophils, contributing to endothelial cell damage, leukocyte aggregation, and systemic inflammation propagation. 

HMGB-1 released by necrotic neurons induces microglia to produce IL-6, resulting in aquaporin water channel expression in astrocytes and exacerbating brain swelling [55]. DAMPs activate the complement cascade, generating complement components C3a and C5a, which in turn trigger complement and inflammatory cells, leading to the release of inflammatory mediators contributing to the systemic inflammatory process. 

During the secondary phase of the brain injury, DAMPSs cause brain edema, initiate secondary vascular damage like microthrombi or vasospasm and result in apoptosis through inflammatory cytokines and microglial shift to an M1 phenotype. DAMPs are cleared via the glymphatic system to peripheral blood and scavenged by various immune cells [56].

### 2.3. Role in Synthesis and Function of Synapses

In addition to providing support to neuronal cells, astrocytes also exist as basic components of the neuronal circuit known as the tripartite synapse and are essential to the structural integrity of synaptic transmission in the brain [57,58,59,60]. Tripartite synapses refer to the integrative communication between the presynaptic neurons, postsynaptic neurons, and the surrounding glial cells [61,62]. In the pathogenesis of altered consciousness or neurodegenerative diseases, astrocytic interactions within tripartite synapses seem to play a role in mood and behavior alterations and the predisposition to neurological diseases [63,64,65].

Astroglia serves a morphological and functional role in these synapses [57,66]. As part of the structural components of the tripartite synapse, astrocytes act as a controller for the active metabolic milieu surrounding neurons [60,67,68,69]. The metabolic milieu in the CNS is important in the formation, maintenance, and function of synapses. Perisynaptic astroglia regulate the CNS milieu through various mechanisms and pathways such as altering extracellular ions, regulating pH, managing waste, and assisting in the exchange of signaling molecules [70,71,72].

Studies have found that under circumstances where astroglia function is compromised, the chemical environment can become neurotoxic and damaging to the surrounding neuronal tissue, thereby negatively affecting synaptic transmission [40,73]. For example, in neurodegenerative diseases such as Alzheimer’s and amyotrophic lateral sclerosis, research suggests that astrocytes are unable to regulate the abnormal accumulation and aggregation of toxic proteins in neuronal tissue, thus leading to an inhibitory milieu, in which hinders the communication between synapses [74,75]. Astroglial cells, including astrocytes, play a pivotal role in the formation and remodeling of synapses. These cells release signaling molecules such as thrombospondin, hevin, neuroligin, and transforming growth factor beta (TGF-β) to promote synaptogenesis of both excitatory and inhibitory neurons [76,77,78,79,80,81,82]. Furthermore, they help dictate the morphology of neuronal dendrite spines, impacting synaptic transmission between neurons [83]. Insults to the brain can result in synaptic damage, and in response, astrocytes aid in synaptogenesis and the restructuring of synapses [84,85]. Studies indicate that astrocytes secrete molecules, including thrombospondin and tumor growth factor beta 1 (TGF-β1), to induce synapse formation [86,87,88]. Beyond synaptogenesis, astroglia also plays a crucial role in neurogenesis. Notably, research has shown that Notch-depleted astrocytes can trigger neurogenesis in injured tissue, which in turn contributes to synaptic plasticity and the restoration of brain function [89,90,91,92,93].

In conjunction with synaptogenesis, astroglial cells can also control synaptic plasticity [94,95]. Astrocytes release neuromediators that induce an increase in the formation of excitatory synapses which are associated with the expression of synaptic plasticity [96]. The induction of synaptic plasticity plays a role in memory formation [97,98]. Contrarily, astrocytes also have the potential to inhibit neural plasticity [99]. Various molecules derived from perisynaptic astrocytes can trigger the elimination and pruning of synapses [100]. Recent studies suggest that synapse regeneration may be stimulated through the manipulation of astrocytic metabolic function in the CNS [101].

Astrocytes are also essential for neurovascular coupling (NVC), which coordinates communication between neurons and blood vessels in the brain. These versatile cells contribute significantly to proper blood vessel density and branching during development, as well as in conditions like ischemic stroke, traumatic spine injury, and by analogy TBI. In response to neurotransmitter release, like glutamate and ATP, astrocytes elevate Ca^2+^ levels, releasing vasoactive molecules onto blood vessels to drive NVC [102].

Astrocytes also dynamically regulate cerebral vessel diameter through Ca^2+^-dependent mechanisms, releasing substances such as EETs (epoxyeicosatrienoic acids) and PGE2 to dilate vessels or 20-HETE (20-hydroxytetraenoic acid) to constrict them. Additionally, they help maintain ion balance by absorbing excess extracellular K+ during neuronal activity, which triggers Ca^2+^ increases, activates BK channels, and releases K+ onto vascular mural cells. Enhanced Ca^2+^ signaling in astrocytes, particularly through metabotropic neurotransmitter receptors, can lead to NVC inversion, where neuronal activity causes vasoconstriction rather than dilation [103].

Astroglia also plays a role in synapse maturation. Several astrocytic-derived genes are associated with proteins that participate in the maturation of synapses and neuronal circuits [104]. For example, activation of surrounding astroglia enhances the expression of postsynaptic density protein 95 (PSD95) and subsequently induces a change in the synaptic organization. Excitatory postsynaptic neurons possess a more diverse nanostructure through the enhanced expression of PSD95 [105].

Perisynaptic astroglia is also an important regulator and modulator of synaptic transmission and function [106,107]. Ref. [108] found that astroglia is involved in inhibitory signaling modulation by interacting with neurons through neuronal cell adhesion molecules (NrCAM). Furthermore, calcium responses in dopamine-activated astrocytes can potentially evoke synaptic depression via adenosine signaling [109,110]. 

Previous studies have found that astroglial cell membranes express a wide variety of diverse molecules that are essential to the regulation of neurotransmission in tripartite synapses [111]. In particular, astroglial cells may express cellular adhesion molecules such as neuroligins, neurexin, and integrins that can influence the interactions between synapses [112,113].

In summation, perisynaptic astrocytes constantly participate in neuronal tripartite synapses through the maintenance of the metabolic milieu, influence on neurovascular coupling, synaptogenesis, and the regulation of synaptic transmission and function. Various mechanisms and pathways are of research interest. Further research into the role of astroglia in synapse formation and function may yield potential medical development toward new treatments for neurodegenerative diseases and disorders of behavior.

### 2.4. Scar Tissue Formation

Reactive astrocytes play a crucial role in isolating brain injuries by forming glial scar tissue around the injury site [114,115,116]. While sealing off injuries provides a protective mechanism, reactive astrogliosis can also hinder brain recovery by blocking axon re-growth and adopting neurotoxic behaviors [117,118,119]. The formation of dense glial scars creates both physical and chemical barriers that obstruct axonal regeneration, potentially leading to further neuronal death [120,121,122,123]. In the wake of neural tissue damage, the immediate response involves inflammation and cell death [124,125,126]. This inflammation then drives the formation of scar tissue through cell proliferation, aiming to replace the damaged tissue [127]. The neural tissue has two mechanisms in which scarring can occur. One of these scarring systems is modulated through fibroblasts [23,128,129]. The other scarring is facilitated by reactive astrocytes in conjunction with reactive microglial cells and glial precursor cells [130]. Astrocytes play an integral part in the support network surrounding neural tissues such as glutamate reuptake, maintenance of the blood-brain barrier, and homeostasis [16,131,132]. Furthermore, reactive astrocytes are important in the facilitation and formation of glial scar tissue [133,134]. In response to neural tissue injury, astrocytes undergo changes in morphology along with hypertrophic changes to their processes [135]. The structure of glial scar tissue consists of a lesion core and the penumbra, otherwise known as the cortical peri-infarct area [24]. Reactive astrocytes form scar tissue in the penumbra region [136,137]. Within the days following injury, the amount of reactive astrocytes increases around the site of the lesion [138]. The mechanism involved in astrogliosis involves the action of ATP on astrocytes [139]. Generally, ATP is released by inflammatory cells after bodily tissue damage. Extracellular release of nucleotides participates in cell signaling and acts on P2X and P2Y purinoceptors [140,141]. P2X is a receptor family belonging to ligand-gated ion channels that produce a quick response to the presence of ATP. On the other hand, P2Y belongs to the G-coupled protein family of receptors that mediates a slower response to ATP [142].

Studies have found the expression of both the P2X and P2Y receptors on the cell surface of astrocytes [143,144]. In the CNS, ATP acts as a neurotransmitter and participates in neuromodulation. Similar to the actions of ATP in other parts of the body, ATP released after neural tissue damage stimulates the P2X and P2Y cell surface receptors that are present in astrocytes in the CNS [145,146]. The extent of neural damage is correlated to the presence of extracellular nucleotides in CSF [147]. These ATP-activated purinergic receptors are shown to be involved in astrogliosis or reactive astrocyte formation and subsequently the formation of scar tissue [148].

**Figure 2:**
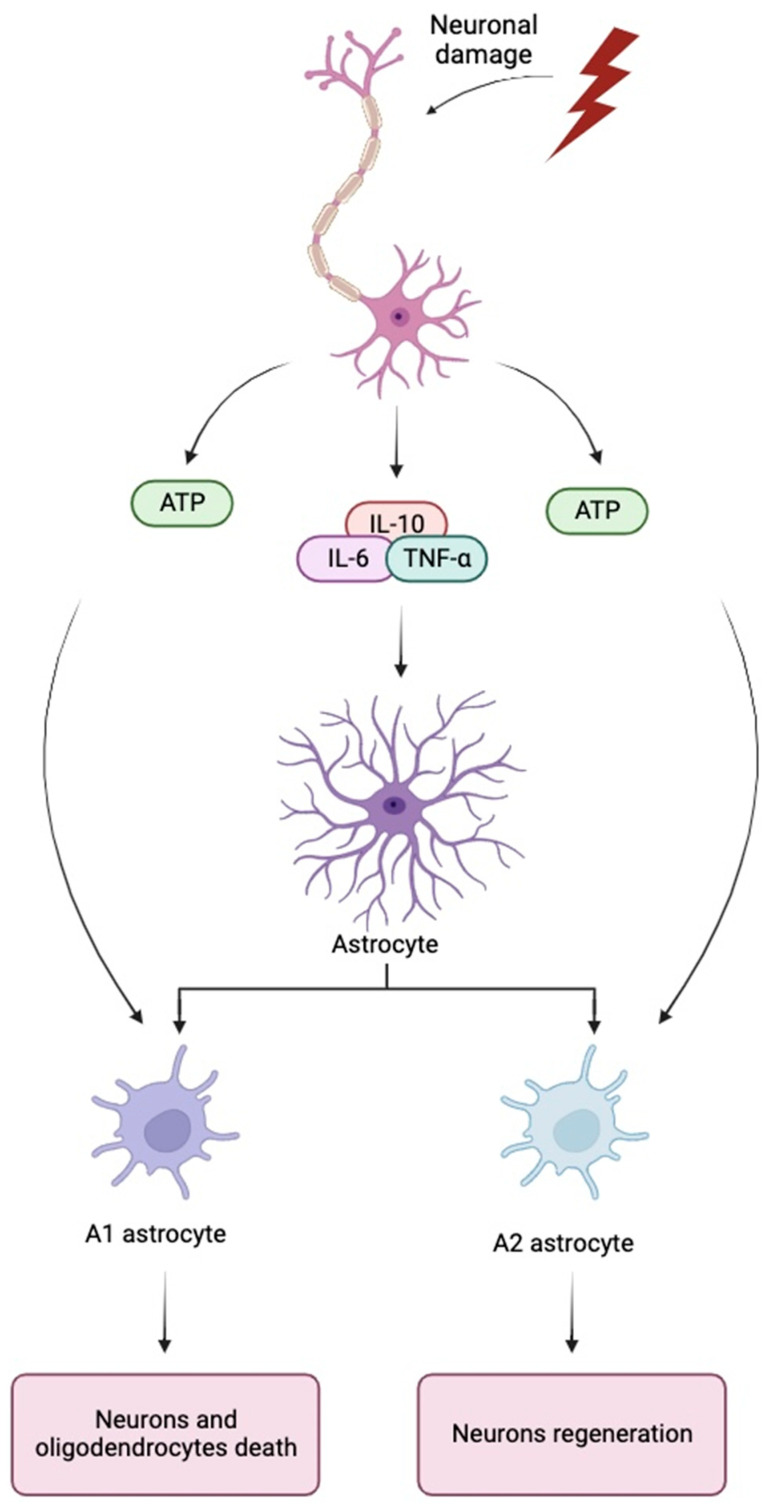
Consequences of stimulation A1 and A2 astrocytes in response to neuronal damage.

There are two types of reactive astrocytes that are important in the formation of neural scar tissues: A1 and A2 [149]. A1 astrocytes stimulate the death of neurons and oligodendrocytes whereas A2 astrocytes are considered neurotropic and neuroregenerative [118,150]. Both A1/A2 astrocytes are stimulated by inflammatory signals released by microglia in response to insults [151,152]. The various types of astrocytes may explain both the inhibitory and protective role of astrocytes during the recovery process after injury [153,154]. Early studies have suggested the formation of glial scar tissue by reactive astrocytes as a healing barrier to the process of axon regeneration within neural tissue [108,155]. Reactive astrocytes are also shown to induce seizures and neuron dysfunction [156,157]. However, as seen in a study by [158], when preventing astrocytes from forming glial scar tissues, the amount of inflammation greatly increased along with an increase in neural degeneration. Further ablation studies have tested the hypothesis that astrocytes also play an aiding role in the regeneration process and shown that the extensive ablation of astrocytes inhibits the regrowth of axons and increased tissue degeneration [159,160]. Findings from these studies have increasingly supported the idea of astrocytes playing both an inhibitory and protective role in the recovery process after CNS injury [161,162] Figure 2.

In its inhibitory role, astrocytes have been associated with inhibition of axonal regeneration [163,164]. Reactive astrocytes produce chondroitin sulfate proteoglycans (CSPG) [165]. After an injury to neural tissue, the expression of CSPG is upregulated and greatly increased [166,167]. Studies have shown that CSPG contributes to the failure in neural tissue regeneration by inhibiting neurite growth [168,169,170]. Therefore, the release of CSPG acts as an inhibitory factor to the migration of cells and axons [171,172,173]. Additionally, other collagens and proteoglycans synthesized by astrocytes such as type IV collagen and NG2 proteoglycan are inhibitory to axon growth as well [172,174].

For normal glial scar formation to occur, intermediate filaments such as glial fibrillary acidic protein (GFAP) and vimentin are needed [175,176]. In the immediate response to neural tissue injury, GFAP is released by reactive astrocytes. Therefore, GFAP can be utilized as a marker for the presence of astrocytes [177,178]. At the site of insult, the amount of GFAP is related to the amount of reactive astroglial cells [179,180]. GFAP is an important intermediate filament that allows astrocytes to become hypertrophic through the synthesis of cytoskeletal structures and elongation of processes [181,182]. An increase in GFAP expression and hypertrophy is evident in the week following injury [183].

Research findings indicate that the absence of insulin receptors (IRs) in astrocytes, specifically in GFAP-expressing astrocytes, results in reduced expression of the vascular endothelial growth factor pathway. IRs in these astrocytes play a crucial role in brain glucose regulation and responses to glucose. They facilitate the entry of circulating insulin into the brain, impacting glucose uptake, brain perfusion, mitochondrial function, and brain vascularization. These receptors are integral to neurovascular coupling, modulating both glucose uptake and vascular function.

The presence of IRs in astroglial end-feet, particularly in GFAP-expressing astrocytes, supports their role in neurovascular coupling and angiogenic signaling. Altered angiogenic signaling in mice lacking astrocytic IRs, specifically in GFAP-expressing astrocytes, may result from changes in mitochondrial function. Interestingly, the extent of the reduction in GFAP-expressing astrocyte IR corresponds to the magnitude of changes in angiogenic signaling. Additionally, IRs in blood-brain barrier cells, including pericytes, influence vessel function [184].

Several cytokines such as IL-10, IL-6, and TNF-ɑ that are produced in response to neural damage may play a regulatory role in the proliferation of astrocytes and thus, the formation of glial scar tissue [185,186,187].

**Figure 3:**
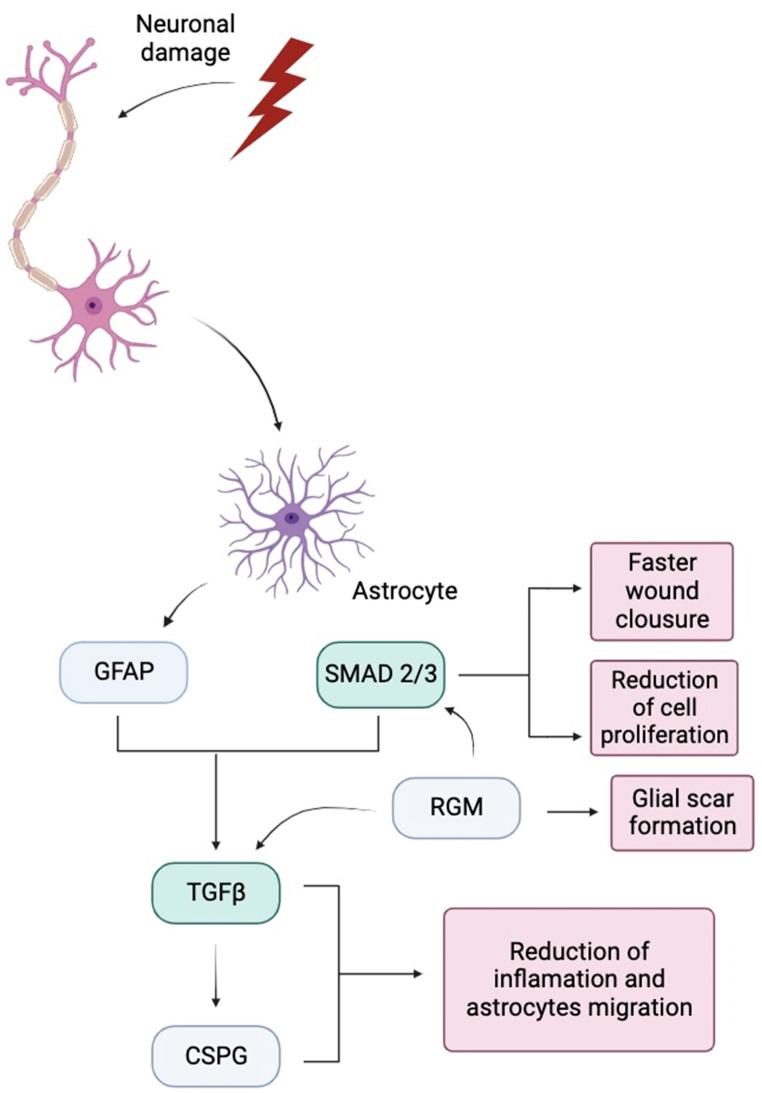
Diagram of astrogliosis induced by TGF-β and Smad 2/3 signaling pathway and its effects.

Another factor that may be important in the activation of astrocytes following injury is the TGF-β and Smad 2/3 signaling pathway [188,189]. Increased expressions of GFAP are associated with the Smad 2/3 signaling pathway which also affects the activation of TGF-β signaling [190,191]. Smad signaling pathway has also shown a reduction in the amount of immune cells and astrocytes present after injury, aiding in faster wound closure and reduction in cell proliferation [192]. Additionally, the relationship between the regulation of Smad 2/ 3 on TGF-β has also been shown to affect CSPG release [191]. Furthermore, studies have shown that may play a role in the reduction in inflammation and astrocyte migration [191,193]. RGMa expression in astrogliosis has also been shown to affect TGF-β and Smad 2/3 signaling and mediation of glial scar formation [194] Figure 3.

During the healing process, damaged scar tissue formed by the reactive astrocytes that have undergone hypertrophy and morphological changes become scar-forming astrocytes [195]. The recruitment of scar-forming astrocytes forms a protective border around inflamed tissue, separating it from healthy neural tissue, and aiding in the recovery process [196,197]. The protective border formed by astrocytes aids in blood-brain barrier repair through the release of ECM components [198]. Scar borders are shown to have been formed from newly proliferated astrocytes that have extended processes [199,200]. These elongated processes have been shown to exhibit overlapping morphology creating a mesh-like network [201]. Astrogliosis and its protective effect are mediated by a transcription factor, STAT3 [202,203]. STAT3 also plays a role in the maturation of glial scars [204] which occurs within weeks after injury [205].

Other beneficial effects include the production of glutathione and ciliary neurotrophic factor (CNTF) by astrocytes. Glutathione plays a protective role for neurons against toxicity from nitric oxide [206,207] whereas CNTF helps support neurons [208,209] which can both increase neuronal survival. 

As studies have shown, astrogliosis plays both an inhibitory and beneficial role in the glial scar formation process through the various components released by astrocytes and the signaling pathways involved. For instance, it has been demonstrated that the activated calcineurin-dependent transcription factor signaling pathway (CN/NFAT) stimulates cytokine production and regulates key metabolic changes of reactive astrocytes. So far, changes in CN/FAT pathway expression in astrocytes have been shown in neurodegenerative conditions animal models such as Alzheimer’s Disease and also in central nervous system injuries [210]. Moreover, astrocytic CN/NFAT inhibition protected neuroplasticity and synaptic function in the TBI rat model, suggesting a potential therapeutic target [211].

### 2.5. Neural Stem Cells and Astrocyte Generation

Radial glial cells (RGCs), which maintain self-renewal and differentiation characteristics and are responsible for the successive creation of neurons, astrocytes, and oligodendrocytes, are generated from neuroepithelial cells of the neural tube throughout the formation of the human CNS. RGCs start directly differentiating into astrocytes or beginning to produce glia-restricted intermediate progenitor cells that can differentiate into astrocytes or oligodendrocyte precursor cells at about 12 post-conceptional weeks in humans [212].

As for the spinal cord, astrocytes are produced in all of its domains following neurogenesis. They migrate laterally along the trajectories of their progenitors following a glial switch and do not migrate tangentially from their domains of origin. A bHLH gene is expressed only in the ventral p2 domain of stem cell leukemia (SCL) and suppresses Olig2 and oligodendrocyte production. The p1, p2, and p3 domains are distinguished by the combinatorial expression of homeodomain proteins Pax6 and Nkx6.1, which are guided by neuronal migration factors such as Slit1 and Reelin in the white matter of the spinal cord. These findings reinforce the notion of region-specific astrocyte subtypes that upon generation stick to coded pathways [213]. 

Adult neural stem cells are located in the dentate gyrus, in the subgranular zone, and lateral ventricles in the subventricular zone, generating new neurons throughout our life [214]. There are distinct differences between these two zones; the dentate gyrus-based neurons stay in their original place becoming interneurons of the olfactory bulb [215], while their LV-generated counterparts’ fate leads them to superficial granule cells and peri-glomerular cells [26].

Mechanically, neural stem cells (NSCs) tend to start their proliferation as adherent cells, forming neurospheres in vitro [216]. Several morphogenic factors have been used to identify forming astrocytes and astrocyte precursors, such as GFAP, SOX9, NF1A, S100β, and MAP–2 [217].

The Sonic hedgehog morphogene-dependent cells that reside in the ventricular zone of the amygdala-hippocampal area during late gestation contribute to the formation of the SGZ in the perinatal stages and are an interesting point for further exploration [218]. 

Signaling pathways and dynamic transcription factor expression trigger transformation in NSCs resulting in gliogenesis [91].

The components involved in these conversions are mentioned: bone morphogenic protein (BMP) families, the leukemia inhibitory factor/ciliary neurotrophic factor (LIF/CNTF), and the Notch pathway [91].

Neurons formed from neural stem cells are undergoing a “gliogenic switch” so that NSCs achieve the ability to generate glial cells—predominantly astrocytes and oligodendrocytes. In turn, the NPCs (neural progenitor cells) convert into glial precursor cells (glioblasts), which through a series of divisions and migrating along radial glia processes, transform into astrocytes. In the last phase of NPCs differentiation by disconnecting radial glia from VZ is formed unipolar transitional radial glia (tRG), which develop into protoplasmic and fibrous astrocytes in the cortex [91].

Research has shown that TGFβ1-mediated induction of nuclear factor IA (NFIA) in tandem with a lengthened G1 phase can promote gliogenesis [219].

In light of this information, ongoing research is trying to mimic natural astrogenesis by inducing human pluripotent STEM cells [217]. One such method was proposed by Peteri et al. to obtain astrocytes with forebrain identity [217].

Steps taken that were necessary for such an outcome were to induce differentiation of hPSC by blocking SMAD signaling. Afterward, the aforementioned SHH and WNT signaling pathways were inhibited using cyclopamine and DKK1, forming proper neural progenitors, expanded in the presence of BDNF, EGF, and bFGF, ready for the glial switch [217]. This model, although efficient, is still unable to provide us with an astrocyte population indistinguishable from the one native to the human CNS, mainly because of the environmental impression on the final stages of astrocyte development [220].

Leventoux et al. created a generation model of a highly homogenous astrocyte population from induced pluripotent stem cells (iPSCs). In this study, an iPSC cell line was derived from a 36-year-old woman. Subsequently, the bone morphogenetic protein inhibitor Dorsomorphin, the TGFβ inhibitor SB431542 and the GSK3β inhibitor CHIR99021 were used, thus iPSCs were unaltered into mesodermal and non-neuronal ectodermal lineages and began the transition into glial and neuronal lines. A subsequent step of this protocol was the transformation into a neuronal line, so for their cultivation from day 7 retinoic acid and purmorphamine were used. The next stage was the multiplication of cells as neurospheres (NS) for 3 weeks. Then with the use of brain-derived neurotrophic factor (BDNF) and glial cell-derived neurotrophic factor (GDNF) tested cells underwent their differentiation into nerve cells, presenting the morphology of both neurons as well as astrocytes. Astrocytes were further isolated to obtain cells with a homogeneous morphology. After an appropriate analysis of marker expression, it was shown that the cells created in this way are immature astrocytes presenting features of functional astrocytes, such as calcium dynamics, neuronal synapse maturation, glutamate uptake activity, and glucose metabolism, which were defined as iPSC-derived astrocytes (iPasts) [221].

It has also been found that post-stroke mice striatal astrocytes also undergo neurogenesis in mice via Notch signaling repression. They abide by similar processes to subventricular zone stem cells, eventually generating new CNS neurons. Research has shown that astrocytes from other parts of the cerebrum, when subject to Notch depletion, behave in a similar way [92].

The Sonic hedgehog (Shh) ligand is crucial during the embryotic ontogenesis of the CNS but also plays an important role in stabilizing the BBB. Shh is produced by astrocytes and promotes the proliferation of adult neural stem cells and oligodendrocyte progenitor cells over the course of many traumatic brain injuries. Shh binds to the Patched receptor (PTC), which in turn activates SMO (Smoothened protein) [222]. This occurs by the GLI transcription factors, expressing GLI1 also known as a glioma-associated oncogene which, when diminished, causes the astrocytes to upregulate GFAP, induce hypertrophy, and proliferation. This phenomenon has been observed to appear in the first 24 h of the injury and linger up to 2 weeks, mostly within the damaged area, and one day may be a useful target for TBI-related research [223].

Taking all of the aforementioned factors into consideration, the research concerning Neural Stem Cells and astrocytes is still far from completion and should be further explored via the proposed methods.

## 3. Differences in Localization, Age, and the Type of Injury

Astrocytes are essential cells in the human CNS, and they are categorized into four main types: protoplasmic, interlaminar, varicose projection, and fibrous [224]. Of significant note, protoplasmic astrocytes are distributed in the grey matter of cortical layers II–VI. They uniquely organize themselves into distinct domains, likely granting them the capability to modulate and synchronize blood flow with synaptic activity [225]. Interlaminar astrocytes, exclusive to primates, project within the cerebral cortex [226,227]. Fibrous astroglia, located near white matter tracts, might interact with neurovasculature [228,229]. Varicose projection astrocytes span specific cortical layers and might play roles in primate cognition [224,230]. Furthermore, there’s compelling evidence pointing to the existence of specialized astrocytes between and within brain regions, paving the way for more in-depth exploration into the varied functions of astrocytes based on their precise location [231]. The exact roles of these subtypes, especially concerning their locations, are still a subject of research [232].

As mentioned above, there is much evidence in the literature that astrocytes are involved in the response to damage to the CNS. However, this response does not always follow the same path and may vary depending on the location and type of damage, as well as the subpopulation of recruited cells.

Studies on the regional differences of reactive astrocytes in the injured cerebral cortex using a cryogenic TBI model have shown that reactive gliosis occurs regionally in a segregated manner in the early stage of injury [233,234].

Regional differences in the glial response were also noted in the case of a cerebral stab wound and a transcranial injury of the spinal cord. In a cerebral stab wound model, GFAP mRNA and GFAP levels were rapidly increased in the proximal astrocytes. Additionally, the proximal astrocytes (closer to the site of damage) exhibited weaker GFAP expression compared to the distal astrocytes, which points to a higher speed of GFAP induction in distal astrocytes [234,235,236,237,238,239].

Localization of reactive astrocytes that display changed morphology depends on the proximity to the insult. Studies have shown that astrocytes close to the injury site have shown greater elongation of processes as compared to astrocytes that are localized further away from the injury site [240]. 

Astrocytes response does not differ only in terms of their location in the CNS. Studies conducted in rodents have also shown differences in astrocytes depending on the type of injury.

The heterogeneous population of reactive astrocytes in the glial scar is most likely due to the ability of NG2 glial cells to differentiate into astrocytes in damaged brain tissue. NG2 glial cells, integral to the adult mammalian CNS, make up 5–8% of its cells and are pivotal in producing myelinating oligodendrocytes and astrocytes. Positioned near neuronal bodies, they receive synaptic inputs and play a role in modulating the neuronal network. Their activity, prominent in early life, diminishes with age, linking them to certain neurodegenerative conditions. They are essential for maintaining a healthy neural environment, with their dysfunction leading to neuronal impairments [241].

Previously, astrocytes have been conventionally regarded as a homogenous cell type [242]. However, recent evidence has revealed an unexpected level of heterogeneity in their roles and development. As a result of TBI, naïve astrocytes are transformed into reactive astrocytes, which regulate neuroinflammation and scar formation [18].

Research indicates that the mode of damage determines the differentiation of the heterogeneous population of astrocytes from the NG2 glium [243]. 

Moreover, in a recent study, Hasel et al. effectively illustrated the diversity of reactive astrocytes in the brains of mouse models induced with LPS [244]. However, differences in type, degree, and location of injury, in vitro conditions, and used animal species make accurate typing of reactive astrocytes more complicated [149]. Nevertheless, unless the precise classification of astrocyte subtypes in the TBI context remains hampered based on available evidence, research on this topic is essential for better understanding the role of astrocytes in TBI pathophysiology [149]. Previous studies demonstrated that reactive astrocytes could clear myelin debris via upregulation of the ABCA1 pathway [245] and enhance but also aggravate BBB integrity [149]. On the other hand, astrocytes with overexpressed AQP-4 are primarily responsible for cytotoxic edema following TBI [246]. Moreover, the downregulation of glutamate transporters GLT-1 and GLAST in astrocytes after TBI enhances their excitotoxicity, leading to aggravation of TBI severity [247].

Numerous cytokines are involved in the changes, both at the local and systemic level, which, in the event of an excessive inflammatory response, cause an autoreactive response against nerve cells. This chronic body response begins when the failure occurs and can last for many weeks [248]. Early-stage glial scar formation is a favorable phenomenon as it isolates the site of injury and potentially dangerous molecules from healthy tissue. Therefore, it is considered to be the body’s protective mechanism. During the acute phase, the most important cells of the immune system are neutrophils, macrophages, microglia, and T-lymphocytes.

On the other hand, in the chronic phase (>14 days after injury), RAs gradually transform into SAs, and astrocytic scars form, which obstructs axonal regeneration. It can be concluded that chronic SA, after the inflammation subsides, is an unfavorable phenomenon. The chronic phase reaches its peak after 60 days and can last up to 180 days [248]. The severity of neural tissue damage also influences the number of reactive astrocytes that are present with more severe injuries requiring the actions of more reactive astrocytes [249]. Additionally, studies have also shown that more severe injuries exhibit more overlapping astrocytic processes [24].

Some biological factors are present in both the acute and chronic phases of glial scar formation. An example is BMP (BMPR1b receptor expression) which plays an important role in the chronic phase as well as during GS stabilization.

## 4. Astrocytes as a Therapeutic Option in TBI

In the field of neuroscience, it is constantly difficult to find therapeutic options for numerous disease entities. For example, there is still a significant challenge in assisting individuals with CNS injuries. There are various approaches to searching for therapeutic methods for people with disabilities. These methods encompass different mechanisms and stages of the condition. Several of them include: gene therapy, pharmacotherapy, neuroprotection, physical intervention, bioengineering, neurorehabilitation, or cell therapy that directly affects cells (including the astrocytes) [250,251,252,253]. The activity of astrocytes in the pathogenesis of traumatic spinal cord and brain injuries is being used as a basis for developing healing methods [149,254]. It has been demonstrated that after injury, astrocytes undergo activation into reactive astrocytes among which can be distinguished two subpopulations: A1 and A2. The A1 population is responsible for pro-inflammatory and neurotoxic mechanisms, whereas A2 acts in an anti-inflammatory and neuroprotective role [145,149,255]. Activated astrocytes release a broad spectrum of substances that influence neurogenesis, synaptogenesis, synaptic stability, and angiogenesis, potentially serving as targets for therapeutic interventions [22]. Notwithstanding, many authors consider this division to be arbitrary at this stage and require further research. Subsequently, they transform into scar-forming astrocytes, and then they undergo another transformation into a fibrous scar. Modifications in the course of this process can serve as a focal point for modern therapies in CNS injuries [149].

Another serious, crippling disorder is TBI, which occurs as a result of the impact of an external force on brain structures. In the case of TBI, we distinguish two phases of damage: primary and secondary. Primary damage in TBI results from the direct impact of the injury on brain tissue. Meanwhile, the secondary phase is the consequence of a series of biochemical and pathophysiological processes that occur as a result of the primary damage, lasting from a few hours to even several years [22,256].

In the secondary phase, there is an inflammatory process taking place. It involves many cells, both of brain cells and originating from the peripheral immune system, among which, notable are: microglial cells, astrocytes, macrophages, leukocytes, monocytes, and neutrophils. Their actions lead to neuronal damage, consequently resulting in neurodegeneration of brain tissue. Similar to spinal cord injuries, gaining a better understanding of the mechanisms during the second phase of brain injury could become a promising focal point for future TBI therapies [22,257]. One such method involves utilizing endocannabinoids present in astrocytes and the associated processes. Endocannabinoids are molecules produced by the body that act as neurotransmitters and exhibit significant anti-inflammatory effects. One of them is 2-arachidonoylglycerol. An analysis was conducted on the impact of the metabolism of this molecule on neurodegenerative processes associated with brain injury. The results demonstrated that increased levels of 2-arachidonoylglycerol in astrocytes, achievable through the inhibition or removal of the enzyme responsible for metabolizing this endocannabinoid namely monoacylglycerol lipase (MAGL), exert a neuroprotective effect in TBI. In mice after TBI MAGL was genetically inactivated, an improvement in cognitive and synaptic functions was observed [257].

Furthermore, another research group carried out trials with the use of 17β-estradiol. The studied group consisted of mice after brain injury. This inquiry proved that 17β-estradiol may have a meaningful role in processes enabling the resumption of proper functioning of the brain. It reduces the expression of neurotoxic astrocyte-activating factors e.g., NF-κ, and also inhibits the release of pro-inflammatory cytokines produced by astrocytes. The clinical effect of these processes is the improvement of neurological functions in mice after TBI [39].

As previously mentioned, astrocytes may play a critical role in safeguarding neural tissue following TBI. They contain Connexin 43 (Cx 43) encoded by the GJA1 gene, which exhibits protective properties against neuronal damage [23,258].

The mechanism of this process has remained unknown until now. It was decided to investigate it more thoroughly. The focus was on the role of GJA1-20K/Cx43 in interactions between astrocytes and neurons. The experiment was conducted on mice. Cortical brain neurons were cultured from C57BL/6 rodent fetuses. Their damage was artificially induced using nitrogen-oxygen mixed gas to mimic natural conditions. Subsequently, a transwell astrocyte-neuron co-culture system was established to replicate the interaction between these cells. It has been demonstrated that overexpression of GJA1-20K enhances the expression of functional Cx43 in astrocytes, facilitating the transfer of mitochondria from astrocytes to neurons. It is speculated that this process may contribute to the formation of protective functions that astrocytes perform towards neurons [23]. Also investigated was how exogenous GJA1-20k affects the apoptosis and mitochondrial function of neurons after TBI. For this purpose, we used exosomes, which are small particles produced by many cells in the body, including astrocytes, containing nucleic acids, proteins, lipids, and metabolites. Astrocytes and neurons were cultured from the rat brain, creating an experimental group—cells were subjected to injury to mimic in vivo TBI, and a control group—healthy nervous system cells. Exosomes were isolated from both the experimental and control groups, noting that their quantity was higher in the damaged cells. The expression of GJA1-20k was also detected, with a higher level observed in cells subjected to TBI. Exosomes were obtained from astrocytes and delivered to the experimental group neurons. It was demonstrated that those with higher GJA1-20k expression, better protected neurons from apoptosis. In damaged neurons, an increase in Cx43 phosphorylation was observed. The delivery of exosomes containing the GJA1-20k gene significantly reduced its level and improved mitochondrial metabolism. Furthermore, GJA1-20k also positively influenced the structure and morphology of mitochondria in post-injury cells. Treatment with exosomes containing GJA1-20k substantially reduced dendritic damage. Additionally, histopathological and histochemical assessment of the rodent brain showed improvement after just one week. Further research into the modification of Connexin 43 and its encoding gene could serve as a desirable target for TBI therapy [258]. It is also worth mentioning the use of Maraviroc in the treatment of TBI. Maraviroc is a C-C chemokine receptor type 5 antagonist, used as a drug in antiretroviral therapy for HIV infection. Its effects in TBI treatment were evaluated, and the results obtained demonstrated that it supports the survival of neurons. Among the mechanisms of action, include regulating microglial differentiation from M1 to M2, reducing neutrophil and macrophage infiltration, inhibiting the release of inflammatory factors, as well as decreasing the activation of previously mentioned pro-inflammatory reactive astrocytes that play a significant role in neurodegeneration after injury. Improvement in cognitive function and motor abilities was observed in the tested mice [145].

Recent research highlights HMGB1 (DAMP) as a key factor in various CNS disorders like Alzheimer’s, Parkinson’s, Multiple Sclerosis, Epilepsy, TBI, and SAH. Targeting HMGB1 has shown neuroprotective effects by inhibiting its expression and release, and reducing inflammatory molecule expression. Common strategies include anti-HMGB1 monoclonal antibodies and natural inhibitors like Glycyrrhizin and Ethyl pyruvate, showing promise in treating CNS and PNS diseases. Moreover, clearing and cleaning up DAMPs from brain injuries appears to be crucial in halting the worsening of lesions, and might represent potential targets for future therapies.

Targeting calcium signaling in reactive astrocytes provides another promising therapy for TBI treatment. Nuclear calcium signaling pathways are instrumental in converting variations in synaptic inputs and neuronal activity into distinct transcriptional patterns. These patterns not only influence the survival of neurons and the upkeep of synaptic connections but also shape the interactions between neurons and glial cells. 

Excessive reactive oxygen species (ROS) and calcium influx at the injury site are primary contributors to secondary injury in TBI. To address this, Zhengzhong Han et al. developed a targeted nanoparticle containing nimodipine (Np) (known as CL-PPS/Np). This nanoparticle inhibits calcium influx in neurons through Np and scavenges ROS in the brain injury environment with poly(propylene sulfide)_60_ (PPS_60_), thus preventing secondary TBI damage [259].

In post-TBI models, CL-PPS/Np accumulates in the injury site and extends the circulation time of Np systemically. It significantly preserves the integrity of the blood-brain barrier, mitigates brain swelling, reduces cell death and inflammation, and enhances functional recovery after brain insult [259].

In a study conducted by A. Fröhlich et al., calcium buffers were introduced in neurons and induced traumatic brain injury (TBI) to study its effects. Surprisingly, buffering neuronal calcium increased microglia recruitment, synaptic loss, and reduced function. Transcriptome analysis revealed complex gene expression changes [260].

Notably, buffering calcium reduced neuronal osteoprotegerin (OPG), while stimulating firing increased OPG. Restoring OPG reduced microglial recruitment and synaptic loss. That leads to an assumption that neuronal calcium signals affect TBI-related neuroinflammation and synaptic loss, with OPG playing a role, impacting functional recovery [260].

There are many potential opportunities for utilizing astrocytes in the therapy of the traumatic brain. Currently, we are already beginning to possess significant foundations for the development of new treatment methods. Nonetheless, primarily for safety reasons, the necessity of a more precise understanding of certain mechanisms and the potential consequences of intervening in them, further work involving astrocyte-based intervention is required. 

## 5. Conclusions

Traumatic Brain Injury persists as a formidable challenge in contemporary medicine, particularly among younger demographics. The absence of specific neuroprotective strategies that significantly affect patient mortality necessitates current interventions focused on mitigating secondary cerebral damage initiated by a cascade of physiological responses to the injury. In this context, astrocytes play a pivotal role through the mechanism of reactive astrogliosis. Nonetheless, research indicates their bidirectional role in CNS injury response. Astrocytes critically influence the function and structural integrity of the blood-brain barrier, affecting its regeneration, ionic equilibrium, and vascular permeability. This is achieved through their interactions with endothelial cells and the secretion of specific cytokines. Additionally, they are integral in maintaining synaptic transmission integrity by modulating the metabolic environment (regulating pH, neurotransmitter exchange, ion metabolism), and secreting synaptogenic factors like thrombospondin, hevin, neuroligin, and transforming growth factor beta (TGF-β). There is also evidence of astrocytes influencing synaptic maturation and functionality, as well as producing neuroprotective factors such as Brain-Derived Neurotrophic Factor (BDNF) and 17β-estradiol (E2). Conversely, astrocytes may exacerbate inflammatory processes, potentially exerting neurotoxic effects on nervous tissues. Their role in glial scar formation is similarly dualistic: it serves as a protective mechanism to isolate damaged areas from healthy tissue, yet it also presents a physical and chemical impediment to regenerative processes, obstructing axonal regeneration, neurite outgrowth, and synaptogenesis.

Interestingly, astrocytic response pathways to damage are contingent upon factors such as injury type, severity, and location. Manipulating these mechanisms could significantly influence patient prognosis post-TBI. Given the complexity inherent in remodeling damaged brain tissue, this presents a formidable challenge. Current research efforts are directed towards modulating astrocytic regenerative functions using agents like endocannabinoids or 17β-estradiol. Promising results have also emerged regarding the use of nimodipine-containing nanoparticles and C-C chemokine receptor type 5 antagonists. However, these findings are primarily derived from animal model studies. While astrocytes present a promising avenue for developing novel treatment modalities for brain injury, further human-based research is imperative.

## Data Availability

Not applicable.
